# Supplementary material for: Analysis of Genetic Diversity and Population Structure of Cowpea (Vigna unguiculata (L.) Walp) Genotypes Using Single Nucleotide Polymorphism Markers
Source: Plants (Basel). 2022 Dec 12;11(24):3480. doi: 10.3390/plants11243480 (PMC9780845; doi:10.3390/plants11243480)
Supplement: Supplementary file 1 [file plants-11-03480-s001.zip › plants-1992921 Supplementary Figure.pdf]

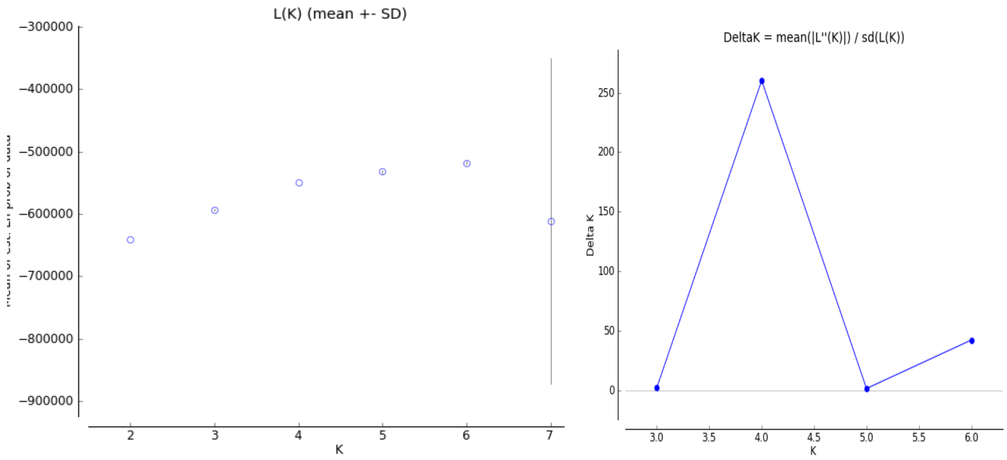

Supplementary Figure S1. Population structure analysis using a Bayesian-based approach. Estimation of hypothetical subpopulations using K-values showing the highest Delta k value was observed at the number of populations (K) = 4.
