# Supplementary material for: Analysis of Genetic Diversity and Population Structure of Cowpea (Vigna unguiculata (L.) Walp) Genotypes Using Single Nucleotide Polymorphism Markers
Source: Plants (Basel). 2022 Dec 12;11(24):3480. doi: 10.3390/plants11243480 (PMC9780845; doi:10.3390/plants11243480)
Supplement: Supplementary file 1 [file plants-11-03480-s001.zip › plants-1992921 Supplementary Table.pdf]

Supplementary Table S1: List of cowpea genotypes used in the study and their origin.

Genotype code	Genotype Code	Accession name	Origin	Growth habit	Seed shape	Seed coat color
**G1**	Acc-Cowp2	ARC002	South Africa	Prostrate	Kidney	Red
**G2**	Acc-Cowp3	ARC003	South Africa	Prostrate	Kidney	Brown
**G3**	Acc-Cowp4	ARC004	South Africa	Prostrate	Kidney	Red
**G4**	Acc-Cowp5	ARC005	South Africa	Prostrate	Rhomboid	Black
**G5**	Acc-Cowp6	ARC006	South Africa	Prostrate	Kidney	Brown
**G6**	Acc-Cowp9	ARC009	South Africa	Semi-erect	Rhomboid	Cream
**G7**	Acc-Cowp10	ARC010	South Africa	Prostrate	Rhomboid	Purple
**G8**	Acc-Cowp11	ARC011	South Africa	Climbing	Kidney	Light brown
**G9**	Acc-Cowp12	ARC012	South Africa	Prostrate	Rhomboid	Brown
**G10**	Acc-Cowp13	ARC013	South Africa	Prostrate	Kidney	Brown
**G11**	Acc-Cowp14	ARC014	South Africa	Erect	Ovoid	Red
**G12**	Acc-Cowp15	ARC015	South Africa	Prostrate	Ovoid	Red
**G13**	Acc-Cowp16	ARC016	South Africa	Prostrate	Rhomboid	Black
**G14**	Acc-Cowp17	ARC017	South Africa	Erect	Ovoid	Cream
**G15**	Acc-Cowp18	ARC018	South Africa	Erect	Rhomboid	Red-speckled
**G16**	Acc-Cowp19	ARC019	South Africa	Erect	Globose	Light brown
**G17**	Acc-Cowp20	ARC020	South Africa	Erect	Rhomboid	Red
**G18**	Acc-Cowp21	ARC021	South Africa	Erect	Kidney	Cream
**G19**	Acc-Cowp22	ARC022	South Africa	Prostrate	Rhomboid	Brown
**G20**	Acc-Cowp23	ARC023	South Africa	Unknown	Rhomboid	Brown
**G21**	Acc-Cowp24	ARC024	South Africa	Climbing	Rhomboid	Black
**G22**	Acc-Cowp25	ARC025	South Africa	Climbing	Rhomboid	Red
**G23**	Acc-Cowp26	ARC026	South Africa	Semi-erect	Kidney	Grey-speckled
**G24**	Acc-Cowp27	ARC027	South Africa	Semi-erect	Kidney	Red-speckled
**G25**	Acc-Cowp28	ARC028	South Africa	Prostrate	Kidney	Red
**G26**	Acc-Cowp29	ARC029	South Africa	Prostrate	Kidney	Brown
**G27**	Acc-Cowp30	ARC030	South Africa	Semi-erect	Globose	Black
**G28**	Acc-Cowp31	ARC031	South Africa	Prostrate	Globose	Cream
**G29**	Acc-Cowp32	ARC032	South Africa	Prostrate	Kidney	Red
**G30**	Acc-Cowp33	ARC033	South Africa	Prostrate	Kidney	Red
**G31**	Acc-Cowp34	ARC034	South Africa	Semi-erect	Rhomboid	Light red
**G32**	Acc-Cowp35	ARC035	South Africa	Climbing	Kidney	Light brown
**G33**	Acc-Cowp36	ARC036	South Africa	Climbing	Kidney	Light brown
**G34**	Acc-Cowp37	ARC037	South Africa	Prostrate	Kidney	Light brown
**G35**	Acc-Cowp38	ARC038	South Africa	Climbing	Globose	Black
**G36**	Acc-Cowp39	ARC039	South Africa	Semi-erect	Kidney	Red
**G37**	Acc-Cowp40	ARC040	South Africa	Semi-erect	Kidney	Black
**G38**	Acc-Cowp41	ARC041	South Africa	Erect	Ovoid	Cream
**G39**	Acc-Cowp42	ARC042	South Africa	Unknown	Kidney	Brown

**G40**	Acc-Cowp43	ARC043	South Africa	Semi-erect	Kidney	Brown
**G41**	Acc-Cowp44	ARC044	South Africa	Erect	Kidney	Light brown
**G42**	Acc-Cowp46	ARC046	South Africa	Erect	Kidney	White
**G43**	Acc-Cowp47	ARC047	South Africa	Erect	Rhomboid	Brown
**G44**	Acc-Cowp48	ARC048	South Africa	Prostrate	Kidney	Cream
**G45**	Acc-Cowp49	ARC049	South Africa	Prostrate	Rhomboid	Brown
**G46**	Acc-Cowp50	ARC050	South Africa	Prostrate	Kidney	Black
**G47**	Acc-Cowp74	98K-5301	South Africa	Erect	Kidney	White
**G48**	Acc-Cowp64	Glenda	South Africa	Semi-erect	Kidney	Brown
**G49**	Acc-Cowp70	TVU13953	South Africa	Prostrate	Kidney	Cream
**G50**	Acc-Cowp62	VegCowDakCream	South Africa	Erect	Kidney	White
**G51**	Acc-Cowp51	CH 14	South Africa	Unknown	Kidney	Brown
**G52**	Acc-Cowp53	BENSOGLA	South Africa	Unknown	Rhomboid	Brown
**G53**	Acc-Cowp52	IT845-2246	Nigeria	Unknown	Rhomboid	Brown
**G54**	Acc-Cowp54	TVU 11424	Nigeria	Unknown	Kidney	Brown
**G55**	Acc-Cowp55	MAKATINI	South Africa	Unknown	Rhomboid	Brown
**G56**	Acc-Cowp56	VULI	Tanzania	Unknown	Kidney	Brown
**G57**	Acc-Cowp57	PAN 311	South Africa	Unknown	Kidney	Brown
**G58**	Acc-Cowp58	NGOJI	South Africa	Unknown	Kidney	Brown
**G59**	Acc-Cowp59	ENBO BUFF	South Africa	Semi-erect	Rhomboid	Brown
**G60**	Acc-Cowp60	IT93K 1294	Nigeria	Erect	Rhomboid	Purple
**G61**	Acc-Cowp61	OUKAWA	South Africa	Prostrate	Kidney	Cream
**G62**	Acc-Cowp63	IT90K-76	Nigeria	Unknown	Rhomboid	Brown
**G63**	Acc-Cowp65	IT96D-602	Nigeria	Erect	Ovoid	Cream
**G64**	Acc-Cowp66	Bechuana White	South Africa	Unknown	Rhomboid	Brown
**G65**	Acc-Cowp67	5431	South Africa	Erect	Rhomboid	Cream
**G66**	Acc-Cowp68	IT90K-59	Nigeria	Unknown	Rhomboid	Brown
**G67**	Acc-Cowp69	FAHARI	Tanzania	Semi-erect	Kidney	Brown
**G68**	Acc-Cowp71	TVU 6345	Nigeria	Semi-erect	Crowder	Brown
**G69**	Acc-Cowp72	ENCORE	South Africa	Unknown	Kidney	Brown
**G70**	Acc-Cowp73	TVU 12637	Nigeria	Unknown	Kidney	Black
**G71**	Acc-Cowp75	IT00K 1217	Nigeria	Unknown	Rhomboid	White
**G72**	Acc-Cowp76	Veg cowpea 1	South Africa	Semi-erect	Kidney	Purple
**G73**	Acc-Cowp77	Veg cowpea 2	South Africa	Semi-erect	Kidney	White
**G74**	Acc-Cowp78	Veg cowpea 3	South Africa	Erect	Kidney	White
**G75**	Acc-Cowp79	MA1	South Africa	Erect	Rhomboid	Purple
**G76**	Acc-Cowp80	IT89U-412	Nigeria	Unknown	Kidney	Black
**G77**	Acc-Cowp81	M346	South Africa	Erect	Rhomboid	Purple
**G78**	Acc-Cowp82	Ukaluleni	South Africa	Prostate	Kidney	Brown
**G79**	Acc-Cowp83	IT89D-349	Nigeria	Erect	Kidney	Brown
**G80**	Acc-Cowp85	Mpenbeni	South Africa	Prostrate	Rhomboid	Cream
**G81**	Acc-Cowp86	2460	South Africa	Prostrate	Kidney	Cream
**G82**	Acc-Cowp87	Vigna Onb	South Africa	Prostrate	Kidney	Purple

**G83**	Acc-Cowp88	MA2	South Africa	Erect	Rhomboid	White
**G84**	Acc-Cowp89	Meter bean piet	South Africa	Prostrate	Kidney	Cream
**G85**	Acc-Cowp91	Chappy	South Africa	Prostrate	Kidney	Cream
**G86**	Acc-Cowp92	M217	South Africa	Erect	Rhomboid	Purple
**G87**	Acc-Cowp93	Tatro mix	Kenya	Prostrate	Rhomboid	Brown
**G88**	Acc-Cowp94	TV778	Nigeria	Erect	Kidney	Cream
**G89**	Acc-Cowp7	New Line 2	South Africa	Unknown	Kidney	Cream
**G90**	Acc-Cowp1	New Line 1	South Africa	Unknown	Kidney	Brown

Note: VegCowDakCream = Vegetable cowpea Dakama Cream
